# The effect of age on the response to the pneumococcal polysaccharide vaccine

**DOI:** 10.1186/1471-2334-10-60

**Published:** 2010-03-10

**Authors:** Hyunju Lee, Moon H Nahm, Kyung-Hyo Kim

**Affiliations:** 1 Department of Pediatrics, School of Medicine, Ewha Womans University, Seoul, Republic of Korea; 2 Center for Vaccine Evaluation and Study, Ewha Medical Research Institute, Ewha Womans University, Seoul, Republic of Korea; 3 Departments of Pathology and Microbiology, University of Alabama at Birmingham, Birmingham, Alabama, USA

## Abstract

**Background:**

*Streptococcus pneumoniae *is a leading cause of morbidity and mortality in the elderly. To prevent invasive pneumococcal diseases, the 23-valent pneumococcal polysaccharide vaccine (PPV) is recommended in subjects over 65 years of age. Although it has been reported to provide approximately 50-80% protection against invasive disease in the general elderly population, there is still controversy as to the effectiveness of the PPV in the elderly.

**Methods:**

To evaluate the immune response to the pneumococcal polysaccharide vaccine in the elderly, samples from young adults and elderly were obtained before and one month after vaccination. The quantitative and qualitative response to the vaccine were measured by the ELISA and opsonophagocytic killing assay for eight vaccine type serotypes (4, 6B, 9V, 14, 18C, 19A, 19F, 23F) and one vaccine-related serotype (6A).

**Results:**

The response to the pneumococcal polysaccharide vaccine showed a similar response between adults and elderly when evaluated by the ELISA, however the functional activity of the antibodies elicited after vaccination were lower in the elderly group for more than half of the serotypes evaluated. In comparison of the antibody needed for 1:8 opsonic titer, more antibodies were needed in the elderly for serotypes Pn 4, 19F, 23F and 6A, suggesting the functional activity of antibody detected by the ELISA was lower in the elderly compared with the adult group for these serotypes. As for subjects with an opsonic titer <8 after vaccination, only one subject each for serotypes Pn 4, 9V and 6A were found in the adult group. However, up to 10 (30.3%) of the subjects did not show opsonic activity after vaccination in the elderly group for serotypes Pn 4, 9V, 14, 19A and 6A.

**Conclusions:**

Although the amount of antibodies elicited were similar between the two age groups, distinct differences in function were noted. This report highlights the importance of a quantitative and qualitative evaluation of the immunogenic response to the PPV in the elderly age group.

**Trial registration:**

This trial is registered with Clinical trials.gov. Registration number NCT00964769

## Background

*Streptococcus pneumoniae *is an important pathogen worldwide causing infection of the respiratory tract, bacteremia, and meningitis and a leading cause of morbidity and mortality in young children and the elderly. To prevent invasive pneumococcal diseases, the 23-valent pneumococcal polysaccharide vaccine (PPV) is recommended in subjects aged 65 years and over [1]. It provides approximately 50-80% protection against invasive disease in the general elderly population [2], although there is still controversy as to the effectiveness of the PPV in the elderly [3,4].

While the effectiveness of vaccine can be demonstrated by clinical studies directly, clinical studies may not be easy to perform. An alternative to clinical studies is to assess the immune responses to vaccine, 'a surrogate of protection'. For the pneumococcal vaccine evaluation, the concentration of type specific antibody measured with enzyme-linked immunosorbent assay (ELISA) has been applied. However, many observations indicate that the antibody's ability to enhance opsonophagocytosis should be the preferred measure of pneumococcal vaccine-induced immunity [5].

In the elderly, the immune response of PPV measured with ELISA is as much as that in young adults [6-9]. However, the response evaluated by the ELISA in the elderly; the previously used 2^nd ^generation ELISA is known to show little specificity due to cross-reaction with contaminants in the capsular polysaccharide (PS), and although the method has gone through improvements [10-13], nonfunctional antibodies can be detected. Also, there is possibility that the elderly may produce pneumococcal antibodies with sufficient avidity to bind capsular PS adsorbed on ELISA plates, but with insufficient avidity to induce opsonophagocytosis [6], result in producing antibodies that are less opsonic than those produced by young adults. But there are few reports of the opsonic function after the vaccine in the elderly [6,14].

Therefore, to determine the immune response in the elderly age group against the PPV, we performed the ELISA and opsonophagocytic killing assay (OPKA) in pre- and postvaccine sera. The response was compared with healthy adults, which the effectiveness of the vaccine in healthy adults is already established [3,4]. The response was evaluated for eight vaccine type serotypes (4, 6B, 9V, 14, 18C, 19A, 19F, 23F) which are prevalent serotypes of invasive diseases and to which the immunogenicity have been studied widely in various age groups after the introduction of the 7-valent protein conjugate pneumococcal vaccine. Also, due to the fact that cross-protection for 6A in PPV vaccinated subjects has been anticipated but not widely proved, the immune response to the vaccine-related serotype 6A was evaluated.

## Methods

### Subjects and Sera Collection

The study group consisted of subjects over 65 years of age and the control group included subjects under the age of 45 years. The subjects in both age groups were healthy volunteers. All subjects had no previous pneumococcal vaccination history and exclusion criteria included asplenia, cancer, liver or renal failure and history of hypersensitivity to vaccine. Vaccinees in both groups were not immunocompromised and this study did not include patients on chemotherapy, steroid or immunomudulating treatment, diabetes mellitus, alcoholism and chronic lung disease. Paired sera were collected from all participants. Prevaccine sera were obtained just before vaccination and postvaccine sera were obtained 4-6 weeks following vaccination. Sera were stored at -70°C until analysis.

The study protocol was approved by the Institutional Review Board and was conducted in accordance with the Declaration of Helsinki and Good Clinical Practice guidelines and written consent was obtained before enrollment.

### Vaccine

All subjects received a single intramuscular injection of 0.5 ml Pneumo23^® ^(Sanofi-Aventis, Lyon France). A 0.5 ml dose of PPV contains 25 micrograms of purified capsular polysaccharide from each of 23 serotypes (1, 2, 3, 4, 5, 6B, 7F, 8, 9N, 9V, 10A, 11A, 12F, 14, 15B, 17F, 18C, 19A, 19F, 20, 22F, 23F and 33F).

### ELISA anti-PS IgG Antibody Concentrations

Anti-pneumococcal (Pn) antibodies against vaccine serotypes 4, 6B, 9V, 14, 18C, 19F and 23F and vaccine-related serotype 6A were measured by 3rd generation ELISA using both C-PS and 22F serotype capsular PS absorption, as previously described [10,13]. Briefly, each well of a 96-well medium binding microtiter plate (Corning Inc., Corning, NY) was coated with 100 μl of a serotype-specific pneumococcal PS antigen (American Type Culture Collection [ATCC], Manassas, Va.) diluted to a predetermined concentration (Serotype 6A PS was a gift from G. Schiffman), and plates were incubated at 37°C for 5 hours in a humidified chamber. The coated plates were washed with 1 × Tris-buffered saline with 0.01% Brij 35 solution. Test sera were preabsorbed with C-PS (Statens Serum Institut, Copenhagen, Denmark) and 22F capsular PS (ATCC), and the reference standard 89-SF (provided by Carl Frasch, Center for Biologics Evaluation and Research, Food and Drug Administration, Bethesda, MD.) was preabsorbed with C-PS. The pre-absorbed sera and 89-SF were serially diluted 2.5-fold in absorption solution and incubated at 25°C for 30 minutes. After incubation, the sera (50 μl) were transferred to the coated microtiter plates, and the plates were incubated for 2 hours at 25°C. The plates were washed 5 times, and 100 μl of diluted alkaline phosphatase-conjugated goat anti-human IgG (Southern Biotech, Birmingham, AL) was added to each well. After 2-hour incubation, the plates were washed 5 times, and 100 μl of substrate solution {diethanolamine (Sigma, St. Louis, MO.) with 1 mg/ml *p*-nitrophenyl phosphate powder (Sigma)} was added to each well. After 2-hour incubation at 25°C, 50 μl of 3 M NaOH was added to all wells to stop the enzyme reaction. The optical density was measured at 405 nm and 690 nm using an ELISA microplate reader. Optical densities were converted to antibody concentrations using the CDC software for pneumococcal ELISA (written by Brian Plikaytis at the Centers for Disease Control and Prevention, Atlanta, GA. It can be downloaded free of charge from http://www.cdc.gov/ncidod/dbmd/bimb/elisa.htm). A detailed protocol can be found at a website http://www.vaccine.uab.edu.

### Opsonophagocytic Killing Assay (OPKA)

The opsonic activities of the samples were evaluated by a double-serotype OPKA, as previously described [15,16]. Briefly, HL-60 cells were differentiated into granulocytic cells by culturing them in RPMI 1640 with 10% fetal calf serum and 0.8% dimethylformamide (Fisher Scientific, Pittsburgh, Pa.) for 5 days. After differentiation, HL-60 cells were diluted to 10^7 ^cells/ml in Hanks' buffer supplemented with 0.1% gelatin and 10% fetal calf serum. Test samples (serum) were also diluted in the same buffer. Target strains expressing capsule type 4, 6A, 6B, 9V, 14, 18C, 19A, 19F, 23F with antibiotic resistance to optochin or streptomycin were reported (~2 × 10^5 ^cfu/ml of each serotype) [15]. Equal volumes of two bacterial suspensions that were chosen to be analyzed together, were pooled. Ten microliters of pneumococcal solution containing 1,000 CFU and 20 μl of test sample were placed in each well of a 96-well microtiter plate. After 30 min of incubation at 25°C, 40 μl of HL-60 cell suspension (4 × 10^5 ^cells per well) and 10 μl of baby rabbit complement (Pelfreeze, Browndeer, Wis.) were added to each well. The mixture was incubated for 1 h at 37°C with shaking. Five microliters of the reaction mixture was plated on two different Todd-Hewitt agar-yeast extract plates. After the fluid was absorbed into the agar, one plate was overlaid with molten Todd-Hewitt agar (0.75%) containing yeast extract (0.5%), 0.5 mg of optochin/liter and 100 mg of TTC (Sigma)/liter [15]. The other plate was overlaid with Todd-Hewitt agar containing yeast extract, 100 mg of streptomycin/liter, and TTC. After an overnight incubation in a candle jar at 37°C, the number of bacterial colonies in the agar plates was enumerated using an automated colony counter. Opsonic titer was defined as the serum dilution that kills 50% of bacteria and was determined by linear interpolation.

### Statisics

Geometric mean concentrations (GMCs) of anti-pneumococcal IgG antibodies, geometric mean titers (GMT) of opsonic titer, geometric mean potency (GMP) for antibody potency and geometric mean indices (GMI) for antibody needed for 1:8 opsonic titer were evaluated and two-sided 95% confidence intervals (CIs) were determined to each pneumococcal serotype. Serum samples with opsonic titer <8 were assigned a value of 4 for analysis purposes. A two-tailed Mann-Whitney U-test was used to analyze differences between pre and post vaccine sera and between age groups. Reverse cumulative distribution curves were used to display percentages of subjects that achieved different opsonic titer or potency to each of the pneumococcal serotypes.

## Results

### Subjects

Thirty-three subjects were enrolled in the elderly group. The age of these subjects ranged from 65 to 70 years (median 66 years). Among these subjects, 20 were male and 13 were female.

The control group consisted of twenty-four adult subjects under the age of 45 years, range from 24 to 37 years. Among these subjects, 13 were male and 11 were female.

### Specific Antibody Concentration by ELISA

The GMCs and 95% CIs for pre- and post-immune anti-Pn IgG concentrations are shown in Table 1. After immunization, the GMC increased significantly in seven out of the eight serotypes evaluated in each age group. Anti-Pn 6A IgG did not increase significantly, which is not included in the PPV.

**Table 1:** Geometric means for antibody concentration and opsonization titers (Elderly, N = 33; Adults, N = 24)

Serotype	Age	Pre-IgG concentration (μg/mL)			Post-IgG concentration (μg/mL)			Pre-Opsonic titer (OT)			Post-Opsonic titer (OT)		
					
		GMC	95% CI	Sig	GMC	95% CI	Sig	GMT	95% CI	Sig	GMT	95% CI	Sig
4	Adult	0.43	0.32-0.59	P < 0.05	***2.21***	1.61-3.05	P < 0.05	17	6-47	NS	***1300***	694-2437	P < 0.05
	Elderly	0.27	0.22-0.34		***1.29***	0.88-1.89		10	5-20		***220***	85-569	
6B	Adult	1.06	0.75-1.50	NS	***6.17***	3.51-10.85	NS	364	104-1270	NS	***8023***	5747-11202	NS
	Elderly	1.17	0.85-1.61		***7.73***	4.13-14.48		175	65-473		***5495***	3823-7898	
9V	Adult	1.08	0.66-1.77	NS	***6.05***	4.22-8.68	NS	504	136-1867	NS	***6380***	3097-13140	P = 0.05
	Elderly	0.71	0.51-0.99		***5.54***	3.62-8.49		229	81-649		***4506***	2654-7649	
14^a^	Adult	3.02	1.56-5.86	NS	***18.7***	9.91-35.29	NS	237	71-795	NS	***6036***	4091-8907	P < 0.05
	Elderly	1.92	1.26-2.93		***10.12***	6.47-15.83		579	192-1748		***4673***	2298-9501	
18C	Adult	1.10	0.72-1.67	NS	***5.8***	3.53-9.52	NS	675	236-1931	NS	***7226***	5167-10105	NS
	Elderly	0.95	0.64-1.42		***6.77***	4.42-10.37		273	104-717		***6698***	4481-10012	
19A	Adult	ND		ND	ND		ND	59	21-170	NS	***2995***	1919-4675	P < 0.05
	Elderly	ND			ND			27	12-58		***556***	252-1227	
19F	Adult	3.28	2.38-4.52	NS	***8.42***	5.42-13.1	NS	40	15-105	NS	***2082***	1409-3075	NS
	Elderly	2.87	2.17-3.81		***7.18***	5.18-9.95		43	19-97		***1183***	759-1843	
23F	Adult	0.60	0.37-0.99	NS	***3.22***	1.91-5.41	NS	485	183-1286	NS	***3101***	2027-4746	NS
	Elderly	0.70	0.48-1.01		***5.8***	3.64-9.23		269	111-653		***3314***	2152-5101	

6A	Adult	1.62	1.19-2.22	P < 0.05	2.39	1.78-3.21	NS	54	17-171	NS	***2097***	1041-4226	P < 0.05
	Elderly	3.25	2.21-4.79		4.01	2.81-5.72		17	9-36		***244***	102-587	

*pre vs post P < 0.05*

^a^For Pn 14 OPKA was done in a limited patients due to lack of sera {Adults (N = 10), Elderly (N = 20)}

In a comparison of GMCs between the elderly and adult groups, preimmune and postimmune GMCs did not show a difference between age groups except for Pn 4 and 6A.

### Antibody Function by OPKA

The GMTs and 95% CIs for pre- and postimmune opsonic titers are shown in Table 1. The GMT increased significantly after immunization for all serotypes in both the adult and elderly groups. OPKA was done in limited patients for serotype Pn 14 due to lack of sera {Adults (N = 10), Elderly (N = 20)}.

In the preimmune sera, no difference in GMT was seen between the elderly and adult age groups. However, after immunization, GMT was higher in the adult group for four out of nine serotypes evaluated; Pn 4, 14, 19A and 6A.

**Figure 1:**
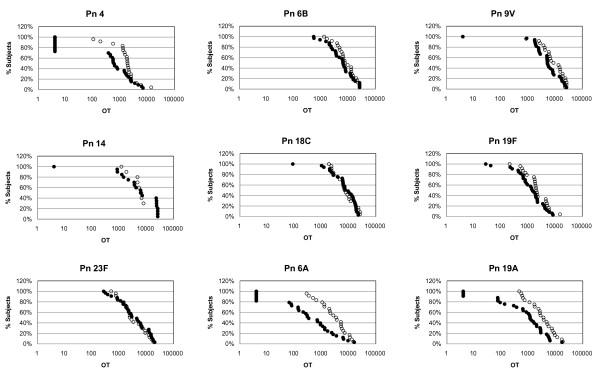
**Reverse cumulative distribution curves for opsonic titers**. Open Circle: Adults; Closed Circle: Elderly. * For Pn 14 OPKA was done in limited patients due to lack of sera {Adults (N = 10), Elderly (N = 20)}.

The reverse cumulative distribution curves for postimmune opsonic titers are shown in Fig. 1 and the data in Table 2 present percentages of subjects who achieved certain opsonic titers to each serotype.

**Table 2:** Subjects with opsonic titer < 8 pre- and post-vaccination

Serotype	Pre-vaccination Opsonic titer<8		Post-vaccination Opsonic titer<8	
		
	Adult (N = 24) n/(%)	Elderly (N = 33) n/(%)	Adult (N = 24) n/(%)	Elderly (N = 33) n/(%)
4	17 (70.8%)	27 (81.8%)	1 (4.2%)	10 (30.3%)
6B	7 (29.2%)	11 (33.3%)	0 (0.0%)	0 (0.0%)
9V	7 (29.2%)	11 (33.3%)	1 (4.2%)	1 (3.0%)
14^a^	7 (29.2%)	7 (21.2%)	0 (0.0%)	2 (6.1%)
18C	4 (16.7%)	8 (24.2%)	0 (0.0%)	0 (0.0%)
19A	11 (45.8%)	17 (51.5%)	0 (0.0%)	4 (12.1%)
19F	12 (50.0%)	14 (42.4%)	0 (0.0%)	0 (0.0%)
23F	3 (12.5%)	7 (21.2%)	0 (0.0%)	0 (0.0%)

6A	12 (50.0%)	21 (63.6%)	1 (4.2%)	7 (21.2%)

^a ^For Pn 14 OPKA was done in a limited patients due to lack of sera {Adults (N = 10), Elderly (N = 20)}.

Before immunization, the number of subjects with no detectable opsonic activity (opsonic titer <8) ranged from 3 (12.5%) for Pn 23F to 17 (70.8%) for Pn 4 in the adult group and 7 (21.2%) for Pn 14 to 27 (81.8%) for Pn 4 in the elderly group. However after vaccination, in the adult group, all subjects showed opsonic activity for serotypes Pn 6B, 14, 18C, 19A, 19F and 23F. Whereas in the elderly group, subjects with no detectable opsonic activity were noted for serotypes Pn 4, 9V, 14, 19A and 6A {range 1 (3.0%) for Pn 9V to 10 (30.3%) for Pn 4}. Number of subjects with no detectable opsonic activity for each serotype is shown in Table 3. In the elderly group, 2 (6.1%) subjects did not show detectable opsonic activity for 3 serotypes and 2 (6.1%) subjects for 2 serotypes.

**Table 3:** Geometric means for antibody potency and antibody needed for 1:8 opsonization index (Elderly, N = 33; Adults, N = 24)

Serotype	Age	Pre-Antibody potency (OT/[IgG])			Post-Antibody potency (OT/[IgG])			Pre-Antibody needed for 1:8 opsonization index (ng/ml)		Post-Antibody needed for 1:8 opsonization index (ng/ml)	
					
		GMP	95% CI	Sig	GMP	95% CI	Sig	GMI	Sig	GMI	Sig
4	Adult	42	16-111	NS	***633***	372-1076	P < 0.05	191.4	NS	***12.6***	P < 0.05
	Elderly	37	18-73		***171***	77-378		218.6		***46.7***	
6B	Adult	343	102-1153	NS	1349	743-2451	NS	23.3	NS	5.9	NS
	Elderly	150	57-392		710	418-1209		53.5		11.3	
9V	Adult	458	145-1448	NS	1112	554-2233	NS	17.5	NS	7.2	NS
	Elderly	324	126-834		813	466-1417		24.7		9.8	
14^a^	Adult	63	23-172	NS	***423***	202-886	NS	126.1	NS	***18.9***	NS
	Elderly	248	71-860		426	211-859		32.3		18.8	
18C	Adult	641	242-1699	NS	1440	951-2182	NS	12.5	NS	5.6	NS
	Elderly	286	121-677		***990***	655-1497		28.0		***8.1***	
19A	Adult	ND		ND	ND		ND	ND	ND	ND	ND
	Elderly	ND			ND			ND			
19F	Adult	12	5-30	NS	***276***	195-391	P < 0.05	653.2	NS	***29.0***	P < 0.05
	Elderly	15	7-31		***165***	128-212		535.7		***48.5***	
23F	Adult	789	309-2012	NS	982	650-1482	P < 0.05	10.1	NS	8.1	P < 0.05
	Elderly	386	170-879		571	396-824		20.7		14.0	

6A	Adult	32	11-96	P < 0.05	***888***	444-1774	P < 0.05	249.1	P < 0.05	***9.0***	P < 0.05
	Elderly	5	3-11		***61***	28-134		1486.5		***131.3***	

*pre vs post P < 0.05*

^a ^For Pn 14 OPKA was done in a limited patients due to lack of sera {Adults (N = 10), Elderly (N = 20)}.

**Figure 2:**
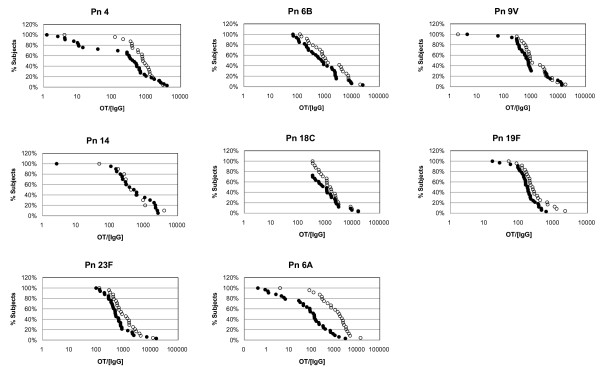
**Reverse cumulative distribution curves for antibody potency**. Open Circle: Adults; Closed Circle: Elderly. * For Pn 14 OPKA was done in limited patients due to lack of sera {Adults (N = 10), Elderly (N = 20)}.

Antibody potency was measured by dividing the opsonic titer by the antibody concentration for each serotype (Table 3) [8]. Although there was no difference in GMP of specific antibody between the two age groups in the preimmune sera (except for Pn 6A), after immunization, antibody potency was significantly higher in the adult group for four out of eight serotypes, including Pn 4, 19F, 23F and 6A. The reverse cumulative distribution curves for the postimmune antibody potency are shown in Fig. 2.

Amount of antibody needed for 1:8 opsonic titer (GMI) was evaluated by dividing the antibody concentration (ng/ml) by the opsonic titer and multiplying by 8 (Table 3) [8]. There was no difference in measurements between the age groups in the pre-vaccine sera. However, after vaccination, GMI was significantly higher in the elderly group for serotypes Pn 4, 19F, 23F and 6A.

## Discussion

After vaccination with PPV, although the amount of antibodies elicited were similar between the two groups, distinct differences in function were noted. After vaccination, the opsonic activity was lower in the elderly group against more than half of the serotypes evaluated. Also, in the analysis for antibody needed for 1:8 opsonization, more antibodies were needed in the elderly for serotypes Pn 4, 19F, 23F and 6A suggesting the functional activity of antibodies detected by the ELISA is lower in the elderly compared with the adult group for these serotypes. Moreover, whereas almost all subjects showed detectable opsonic activity after vaccination in the adult group, up to 10 (30.3%) of the subjects did not show opsonic activity in the elderly group for serotypes Pn 4, 9V, 14, 19A and 6A

Recently, Schenkein et al. reported the opsonic antibody response to the PPV in the elderly [14]. In the article, the opsonic titer and antibody potency showed a significant difference between young and old adults for all 7 serotypes (Pn 4, 6B, 9V, 14, 18C, 19F and 23F) evaluated. Our results showed functional differences for 6 out of 9 serotypes evaluated (Pn 4, 14, 19A, 19F, 23F and 6A). The fact that the elderly subjects in our study were fairly younger (range 65-70 years, median 66 years) could be an explanation for difference in results. Functional decrease in the activity of antibodies has been reported to be more pronounced in the elderly as age increased [6]. Also in previous reports, the antibody titer after vaccination with PPV was better in male elderly compared with females [7] and the majority of the elderly subjects in our study were male.

The prevaccine antibody concentration and opsonic titer were similar between the age groups. Also the antibody needed for 1:8 opsonic titer was similar in the prevaccine sera of the elderly and adults. This is interesting considering rates and mortality from pneumococcal diseases are higher in the elderly group. This could be due to the fact that the subjects in this study were 65-70 years (median 66 years) of age. However, the susceptibility of elderly persons is multifactorial and nonimmunologic factors such as suppression of gag reflex, underlying disease predominate, with chronic cardiopulmonary and other diseases contribute to this. Romero-Steiner et al. reported prevaccination opsonic titers of elderly recipients to be lower when compared with younger adults, where the subjects ranged from age 63-103 years (mean 85.5 years) [6], but the control group consisted of a limited number of subjects (n = 12). Further research on older aged elderly subjects would be desirable.

The results of our study reflect the effect of aging on the B-cell immune system. In the elderly, the B cell production rate decreases, however the number of peripheral B cells remain constant [17,18]. This is due to the capacity of B cell for peripheral self-renewal and their increased life span when new B cells are not produced. However, whereas the magnitude of antibody response to T-independent antigens is maintained during aging, the quality of these antibodies alters during aging [19]. The change of affinity results from compromised selection of high affinity antibodies within the germinal center [19,20].

In this study, we evaluated the response to the vaccine-related serotype Pn 6A. The prevaccine anti-Pn 6A antibody concentration seemed to be higher in the elderly, however after vaccination, the opsonic titer was significantly higher in the adult group. Also, the antibody needed for 1:8 opsonic titer was more than 14 times higher in the elderly group. Although a cross-reactive immune response was elicited to Pn 6A in both groups, a much weaker response was seen in the elderly. Pn 6A is not included in the PPV. Only Pn 6B was included in the PPV because antibodies to the two serotypes were thought to be highly cross-reactive, and Pn 6B is a more stable antigen [21]. The striking difference between age groups in immunogenic response to Pn 6A was an interesting finding which suggests with vaccination of PPV in the elderly, the effectiveness towards pneumococcal diseases by serotype Pn 6A might be lower compared with the adults. Also, recently a new serotype Pn 6C has been discovered and considering reports on increase of Pn 6C in all age groups after routine immunization in children of the 7-valent pneumococcal vaccine are accumulating, close monitoring on the epidemiologic changes of these serotypes in the elderly is desirable [22-24].

Previous reports on the immunogenicity to the PPV in the elderly group show adequate mean immune responses, good antibody fold increase and comparable IgG antibody avidity with adults [7,8,25-27]. These reports are based on immunogenicity evaluated by ELISA. However, clinical data show various results related to the effectiveness of the vaccine, which leads us to acknowledge the need for additional methods of evaluation of the response to the vaccine.

Discrepancy between antibody function and antibody concentration in a clinical setting has been reported [6]. A passive-protection experiment in mice with *S. pneumonia *compared protection from two different sera with the same antibody concentration but different opsonic titers and antibody avidity. The serum with high opsonic titer and antibody avidity showed 100% protection whereas no protection was observed with a serum with the same antibody concentration but low opsonic titer and antibody avidity. These results emphasize the importance of evaluation of antibody concentration and antibody function. Although the OPKA has been known as a labor intensive method, OPKA methods have been evolving rapidly and is now capable of evaluating multiple serotypes for many samples with only small amounts of sera [16,28-31].

This report highlights the importance of a quantitative and qualitative evaluation in this age group. There was a definite overall increase in opsonic titers after vaccination for all serotypes in the elderly, thus the recommendations for vaccination in the elderly should not be discouraged. Also, recently many countries have shown the cost-effectiveness of routine vaccination of PPV in the elderly [32,33]. However, due to the fact that the response in the elderly was weaker for some serotypes and individuals compared with adults, we emphasize the need of evaluation of functional activity within this age group when determining the immunogenic response. Also, with the vigorous development of pneumococcal vaccines, efforts to enhance this weaker response in this age group are warranted. Therefore, the results of this article could be invaluable in the development of the optimal vaccine and making decisions for the proper policy for pneumococcal vaccination in the elderly.

## Conclusions

After vaccination with PPV, although the amount of antibodies elicited were similar between the two groups, distinct differences in function were noted. The immunogenic response to the PPV vaccine should be evaluated by the quantitative and qualitative response in this age group.

## List of abbreviations used

PPV: 23-valent pneumococcal polysaccharide vaccine; ELISA: enzyme-linked immunosorbent assay; PS: polysaccharide; OPKA: opsonophagocytic killing assay; Pn: pneumococcal; GMC: Geometric mean concentration; GMT: geometric mean titer; GMP: geometric mean potency; GMI: geometric mean index; CI: 95% confidence interval;

## Competing interests

The authors declare that they have no competing interests.

## Authors' contributions

HL performed the primary statistical analysis, and wrote the bulk of the paper. KHK performed the laboratory assays and was the principal investigator for the study, reviewed and approved the analyses and contributed to the writing and revision of the paper. MHN supervised the laboratory work, reviewed and approved the analyses and contributed to the writing and revision of the paper. All authors read and approved the final version of the manuscript.

## Pre-publication history

The pre-publication history for this paper can be accessed here:

http://www.biomedcentral.com/1471-2334/10/60/prepub
